# Prediction of Critical Power and W′ in Hypoxia: Application to Work-Balance Modelling

**DOI:** 10.3389/fphys.2017.00180

**Published:** 2017-03-23

**Authors:** Nathan E. Townsend, David S. Nichols, Philip F. Skiba, Sebastien Racinais, Julien D. Périard

**Affiliations:** ^1^Athlete Health and Performance Centre, Aspetar Orthopaedic and Sports Medicine HospitalDoha, Qatar; ^2^Department of Sports Medicine, Advocate Lutheran General HospitalPark Ridge, IL, USA

**Keywords:** high-intensity intermittent exercise, cycling, altitude, hypoxia, fatigue

## Abstract

**Purpose:** Develop a prediction equation for critical power (CP) and work above CP (W′) in hypoxia for use in the work-balance (${\text{W}}_{\text{BAL}}^{\prime }$) model.

**Methods:** Nine trained male cyclists completed cycling time trials (TT; 12, 7, and 3 min) to determine CP and W′ at five altitudes (250, 1,250, 2,250, 3,250, and 4,250 m). Least squares regression was used to predict CP and W′ at altitude. A high-intensity intermittent test (HIIT) was performed at 250 and 2,250 m. Actual and predicted CP and W′ were used to compute W′ during HIIT using differential (${\text{W}}_{\text{BALdiff}}^{\prime }$) and integral (${\text{W}}_{\text{BALint}}^{\prime }$) forms of the ${\text{W}}_{\text{BAL}}^{\prime }$ model.

**Results:** CP decreased at altitude (*P* < 0.001) as described by 3rd order polynomial function (*R*^2^ = 0.99). W′ decreased at 4,250 m only (*P* < 0.001). A double-linear function characterized the effect of altitude on W′ (*R*^2^ = 0.99). There was no significant effect of parameter input (actual vs. predicted CP and W′) on modelled ${\text{W}}_{\text{BAL}}^{\prime }$ at 2,250 m (*P* = 0.24). ${\text{W}}_{\text{BALdiff}}^{\prime }$ returned higher values than ${\text{W}}_{\text{BALint}}^{\prime }$ throughout HIIT (*P* < 0.001). During HIIT, ${\text{W}}_{\text{BALdiff}}^{\prime }$ was not different to 0 kJ at completion, at 250 m (0.7 ± 2.0 kJ; *P* = 0.33) and 2,250 m (−1.3 ± 3.5 kJ; *P* = 0.30). However, ${\text{W}}_{\text{BALint}}^{\prime }$ was lower than 0 kJ at 250 m (−0.9 ± 1.3 kJ; *P* = 0.058) and 2,250 m (−2.8 ± 2.8 kJ; *P* = 0.02).

**Conclusion:** The altitude prediction equations for CP and W′ developed in this study are suitable for use with the ${\text{W}}_{\text{BAL}}^{\prime }$ model in acute hypoxia. This enables the application of ${\text{W}}_{\text{BAL}}^{\prime }$ modelling to training prescription and competition analysis at altitude.

## Introduction

The critical power (CP) concept was originally introduced by Monod and Scherrer (1965), and describes the relationship between sustainable power output and duration for severe-intensity exercise. A simple hyperbolic, two parameter model was proposed:

(1) $$\begin{array}{r} \text{W}\prime ={\text{t}}_{\text{lim}}/\left(\text{P}-\text{CP}\right) \end{array}$$

where W′ = total work accumulated above CP until task failure, t_lim_ = duration until task failure, P = power output, and CP = critical power, defined as a rate limited sustainable power output below which no net expenditure of W′ occurs. Equation (1) can be conceptualized according to a hydraulic model (Margaria, 1976; Morton, 2006), whereby the value W′ progressively depletes during exercise whenever P > CP and reconstitutes when P<CP. Additionally, it can also be seen that if W′ depletes to zero, then t_lim_ also reaches zero and hence task failure is attained. A continuous integral function has been proposed, termed the “work-balance” (${\text{W}}_{\text{BAL}}^{\prime }$) model, which mathematically describes the depletion and reconstitution of W′ (Skiba et al., 2012, 2014a, 2015). A differential equation form of the ${\text{W}}_{\text{BAL}}^{\prime }$ has also been proposed (Skiba et al., 2015). As per the abovementioned hydraulic model, the ${\text{W}}_{\text{BAL}}^{\prime }$ models predict that task failure during an intermittent task is attained when W' depletes to 0 kJ. Both versions of the ${\text{W}}_{\text{BAL}}^{\prime }$ model require CP and W′ as input variables, hence accurate estimation of CP and W′ is a prerequisite for computing ${\text{W}}_{\text{BAL}}^{\prime }$. However, studies have reported that moderate hypoxia decreases CP (Dekerle et al., 2012; Simpson et al., 2015; Shearman et al., 2016) and severe hypoxia decreases both CP and W′ (Valli et al., 2011). Since it is impractical to measure CP and W′ at all possible altitudes, a prediction model which corrects CP and W′ for the effect of hypoxia would permit ${\text{W}}_{\text{BAL}}^{\prime }$ computation at any given altitude.

The mechanistic basis of the two parameter CP model has been extensively studied (Jones et al., 2010). Numerous investigations have demonstrated that CP corresponds to the highest exercise intensity at which pulmonary $\dot{\text{V}}$O_2_ (Poole et al., 1988; Jones et al., 2008; Vanhatalo et al., 2016), and intramuscular high energy phosphates (Jones et al., 2008; Chidnok et al., 2013), can achieve a steady state. The achievement of steady-state $\dot{\text{V}}$O_2_ kinetics indicates the net energetic demand of the task can be met by oxidative metabolism (Poole et al., 2016). When the task requirement increases beyond CP the additional energetic demand is supplemented by substrate level phosphorylation (Vanhatalo et al., 2016) which induces non-steady state $\dot{\text{V}}$O_2_ kinetics, accelerated degradation of high energy phosphates, and accumulation of metabolites involved in peripheral muscle fatigue (Jones et al., 2008; Poole et al., 2016). Since the total amount of work accumulated above CP is terminated at the moment of task failure, the mechanisms leading to task failure itself also determine the value of W′. Recent work examining the etiology of neuromuscular fatigue during high intensity exercise reveals that some combination of both central and peripheral fatigue mechanisms is always present at the moment of task failure, whilst the relative contribution of each depends on the task (Hureau et al., 2016). Observed values for [PCr], [P_i_], and [H^+^] at task failure have been shown to remain similar despite manipulations in pacing strategy during continuous exercise (Burnley et al., 2010), or recovery duration (Chidnok et al., 2012) and recovery power (Chidnok et al., 2013) during intermittent exercise. Also, following exhaustive high-intensity exercise the magnitude of peripheral fatigue, assessed via twitch interpolation, remains consistent in normoxia and moderate hypoxia (Amann et al., 2006b; Romer et al., 2007). Recently it was demonstrated that changes in peripheral fatigue (assessed via twitch potentiation) was significantly correlated to changes in both [P_i_] and [H^+^] during exercise when lower limb muscle afferent feedback was impaired using lumbar intrathecal fentanyl (Blain et al., 2016). Collectively, these findings have led to the theory that within a given task, peripheral muscle fatigue may be regulated via group III/IV afferent feedback, which limits central motor drive to the locomotor muscle (Hureau et al., 2016). The existence of such a feedback loop might explain why W′ appears to resemble a fixed capacity within a given task (Broxterman et al., 2015). From a mathematical perspective, a fixed value of W′ allows performance during high-intensity tasks to be predicted using the 2-parameter CP model.

During exercise in hypoxia, convective O_2_ transport to the working muscle is reduced (Amann and Calbet, 2008), and multiple studies have reported a significant decrease in CP without a corresponding change in W′ (Dekerle et al., 2012; Simpson et al., 2015; Shearman et al., 2016). If CP is lower in hypoxia, then according to the CP model a given absolute exercise intensity in the severe domain will result in a faster rate of W′ depletion. Previously we reported a large error in modelled ${\text{W}}_{\text{BAL}}^{\prime }$ during intermittent exercise performed in hypoxia when the normoxic CP estimate is used (Shearman et al., 2016). Therefore, CP must either be tested in hypoxia, or estimated from measurements in normoxia. Various studies have examined the effect of increasing altitude on $\dot{\text{V}}$O_2max_ reporting either a linear decrease (Wehrlin and Hallén, 2006; Clark et al., 2007), a curvilinear decrease (Péronnet et al., 1991; Bassett et al., 1999), or a curvilinear interaction between altitude and sea level $\dot{\text{V}}$O_2max_ (MacInnis et al., 2015). To our knowledge, no studies have examined the dose-response effect of increasing altitude on CP and W′. Moreover, a large reduction in W′ was reported at high altitude (5,050 m) (Valli et al., 2011), whereas another study found no change at simulated altitude equivalent to 3,800 m (Simpson et al., 2015). Hence, the approximate threshold altitude where W′ begins to decline remains unclear. The purpose of this study was to examine the dose-response effect of increasing altitude on both CP and W′, and thereafter to develop a prediction equation enabling ${\text{W}}_{\text{BAL}}^{\prime }$ computation in hypoxia. A secondary aim was to compare the integral vs. the differential equation form of the ${\text{W}}_{\text{BAL}}^{\prime }$ model. We hypothesized that CP would decline in a curvilinear fashion commencing from the lowest altitude above sea level (1,250 m) tested, whereas W′ would only begin to decline at altitudes above ≈3,800 m.

## Methods

### Participants

Nine trained male cyclists (mean ± SD; age 34 ± 6 year, 78.1 ± 8.0 kg; $\dot{\text{V}}$O_2peak_ 4.57 ± 0.47 L.min^−1^) volunteered to participate in this study, which was approved by the Anti-Doping Lab Qatar Institutional Review Board. All procedures conformed to the standards of the Declaration of Helsinki. Participant inclusion was based on age (18–40 years), training history (2 year minimum cycling training history, 7 h.wk^−1^ minimum average training), and health status (free from injury or illness). All participants were experienced at conducting cycling time trials. Written informed consent was obtained following explanation of the experimental procedures, associated risks, and potential benefits.

### Experimental overview

Participants completed a total of eight testing sessions over a period of 2 months. A minimum of 2, and a maximum of 14 days was specified between any two consecutive lab visits, however, two participants completed one lab visit each outside of this window due to unavoidable personal commitments. The first visit to laboratory involved an $\dot{\text{V}}$O_2peak_ ramp incremental test (30 W.min^−1^) for subject characteristics, followed by a 30 min recovery period and then a 7 min familiarization time trial (TT). Thereafter, on five separate lab visits, participants completed TT's to determine CP and W′ at the following target FiO_2_: 0.203, 0.18, 0.159, 0.14, and 0.123, which corresponds to simulated altitudes of 250, 1,250, 2,250, 3,250, and 4,250 m, respectively. The order of condition was counterbalanced according to a latin square design, with participants blinded to the experimental condition. On the remaining two visits, a HIIT at 250 m and 2,250 m was completed. These sessions were not performed after completion of all five TT testing sessions, but rather on the next lab visit immediately following the TT testing at the same altitude. We chose this experimental protocol to minimize the effect of either training or altitude acclimation, on performance during the HIIT. Participants were instructed to avoid strenuous exercise for 24 h prior to each testing session, and to abstain from caffeine and alcohol on the day of testing.

### Equipment and measures

All exercise tests were performed on an electronically braked cycle ergometer (Schoberer Rad Messtechnik, Jülich, Germany) with power was measured at 1 Hz. All simulated altitude conditions were conducted inside a temperature controlled (20°C) altitude chamber (LoxyMed, Berlin, Germany) with stability of target altitude within ±100 m.

### Critical power testing

The CP test was equivalent to that described and validated by Karsten et al. (2014, 2016). This protocol consists of three TT efforts lasting 12, 7, and 3 min in descending order, interspersed with 30 min of active recovery. We chose to use TTs rather than time to exhaustion (TTe) tests on the grounds that TTs exhibit lower typical error than TTe tests (Paton and Hopkins, 2001) and secondly, recent evidence suggests that CP and W′ parameters estimated from self-paced TTs lead to better prediction of actual TT performance duration then parameters estimated from constant load trials (Black et al., 2015). Participants were blinded to power output, but not duration. Upon completion of the 12 and 7 min TTs, participants exited the altitude chamber within 1-2 min so the first 20 min of the recovery period was always conducted in normoxia. The last 10 min of recovery was conducted inside the chamber at the simulated altitude as specified by the experimental condition.

CP and W′ were initially modelled using three versions of the 2-parameter CP model (1) linear 1/time model, (2) linear work-time model, and (3) nonlinear hyperbolic model (Jones et al., 2008). In each case the standard error of the estimate (SEE) was determined for CP and W′. The lowest SEE for the majority of tests occurred for the linear 1/time model. Therefore, all data analysis used estimates from this model.

### High-intensity intermittent test (HIIT)

The HIIT consisted of nine discreet work intervals performed at a target power output predicted to produce task failure during constant load exercise in 5 min according to the 2-parameter CP model:

(2) $$\begin{array}{r} {\text{P}}_{5}=\left(\text{W}\prime /{\text{t}}_{\text{desired}}\right)\;+\text{ CP } \end{array}$$

Where P_5_ is power output and t_desired_ is the desired time to task failure (300 s). Interval duration ranged from 40 to 60 s and recovery duration from 30 to 60 s. Immediately following every 3rd work interval a maximal sprint effort (3–5 s) was performed in isokinetic mode at 100 rev.min^−1^. After the 9th work interval, there was a 2.5 min recovery period followed by a self-paced, maximal effort 3 min TT (3TT). Power during all recovery periods was 60 W.

### Altitude prediction and ${\text{W}}_{\text{BAL}}^{\prime }$ modelling

Mean CP and W′ estimates from each altitude were expressed as a percentage of the values obtained during testing at 250 m. These values were fitted to a 3rd order polynomial using ordinary least squares regression (GraphPad PRISM, USA). Change in W′ with increasing altitude was described using a two-segment linear regression approach since it was expected that no change would occur in W′ until the highest altitude tested (Valli et al., 2011; Dekerle et al., 2012; Simpson et al., 2015; Shearman et al., 2016). Slope one was constrained to 0 (%.km^−1^) and intercept one was constrained to 100% of baseline level (250 m). Breakpoint, slope and intercept two were left unconstrained. Only measured values for CP and W′ were used to model ${\text{W}}_{\text{BAL}}^{\prime }$ during the intermittent task at 250 m, whereas both the actual measures of CP and W′, and corrected values based on the prediction models, were used to compute ${\text{W}}_{\text{BAL}}^{\prime }$ at 2,250 m.

Modelling of ${\text{W}}_{\text{BAL}}^{\prime }$ during HIIT was conducted using two different equations referred to as the “integral” (${\text{W}}_{\text{BALint}}^{\prime }$) model (Skiba et al., 2012) and the “differential” (${\text{W}}_{\text{BALdiff}}^{\prime }$) model (Skiba et al., 2015). A detailed mathematical derivation from ${\text{W}}_{\text{BALdiff}}^{\prime }$ to ${\text{W}}_{\text{BALint}}^{\prime }$ can be found in the appendix section of Skiba et al. (Skiba et al., 2015). Briefly, the ${\text{W}}_{\text{BALint}}^{\prime }$ model deducts cumulative work expended (or recovered) from the initial W′ to determine ${\text{W}}_{\text{BAL}}^{\prime }$ remaining during an intermittent task. The discharge and reconstitution rate of ${\text{W}}_{\text{BAL}}^{\prime }$ occurs exponentially as shown in Equation (3):

(3) WBALint′=W′−∫0tW′exp · e−(t−u)Wτ′ · du

Where ${\text{W}}_{\exp}^{\prime }$ is the amount of W′ presently expended, and (t-u) is equal to the time in seconds where the athlete is recovering below CP. The time constant for the reconstitution of W′ ($\tau_{{\text{W}}^{\prime }}$) is a function of the difference between the recovery power and the individual's CP (D_CP_) according to the following equation (Skiba et al., 2012):

(4) $$\tau_{\text{W}\prime }=\text{546 }\cdot {\text{ e}}^{\left(-0.01{\text{D}}_{\text{CP}}\right)}+316$$

The ${\text{W}}_{\text{BALdiff}}^{\prime }$ model treats W′ as a chemical reactant. As per the integral form, W′ reconstitution follows an exponential time course, whilst discharge is strictly linear. However, the time constant is calculated by dividing the starting W′ by D_CP_ rather than fitting data as per equation 4. The discharge of W′ when P>CP using the differential form of the ${\text{W}}_{\text{BAL}}^{\prime }$ is given by:

(5) $${\text{W}}_{\text{BALdiff}}^{\prime }={\text{W}}_{0}^{\prime }-\left({\text{W}}_{0}^{\prime }-\text{W}\prime \left(u\right)\right)e^{-\frac{{\text{D}}_{\text{CP}}}{{\text{W}}_{0}^{\prime }\left(\text{t}-\text{u}\right)}}$$

Where ${\text{W}}_{0}^{\prime }$ is the initial starting value of W′ prior to a work segment where P>CP, and as above (t-u) is equal to the segment of time where P>CP. Recovery of ${\text{W}}_{\text{BALdiff}}^{\prime }$ occurs during a segment of time when P<CP according to Equation (6):

(6) $${\text{W}}_{\text{BALdiff}}^{\prime }={\text{W}}_{0}^{\prime }-{\text{W}}_{exp}^{\prime }\;e^{-D_{CP}t/W_{0}^{\prime }}$$

Where ${\text{W}}_{\exp}^{\prime }$ is the W′ expended during the prior segment in which P>CP. The time course for the entire HIIT is computed by sequentially determining depletion and recovery for each successive segment, where P>CP and P<CP, respectively.

Modelled ${\text{W}}_{\text{BAL}}^{\prime }$ for both the integral and differential equations was computed at 1 Hz throughout the HIIT, but only values at completion of each interval (1 through 9), and the final 3TT, are reported.

### Statistical analysis

Statistical analysis was completed on all data using the Statistical Package for Social Sciences (SPSS) Version 22.0 (SPSS Inc., Champaign, IL). Normality of the data was checked using the Shapiro-Wilk test with (*P* < 0.05) indicating non-normality. Linear mixed modelling was used to examine the fixed effect of altitude on CP and W′, and also to examine fixed effects of model (${\text{W}}_{\text{BALint}}^{\prime }$ vs. ${\text{W}}_{\text{BALdiff}}^{\prime }$), parameter input (actual vs. altitude corrected CP), altitude (250 vs. 2,250 m), and interval (1 to 9 + 3TT), on modelled ${\text{W}}_{\text{BAL}}^{\prime }$. Random effects were designated as participant slope and intercept. *Post-hoc* pairwise comparisons were conducted using Sidak's correction and effect sizes were calculated using Hedges' *g*. All pairwise comparisons are reported as mean difference (95% confidence interval: lower, upper; hedges' *g*; *P*-value).

## Results

### Effect of altitude on CP and W′

**Figure 1:**
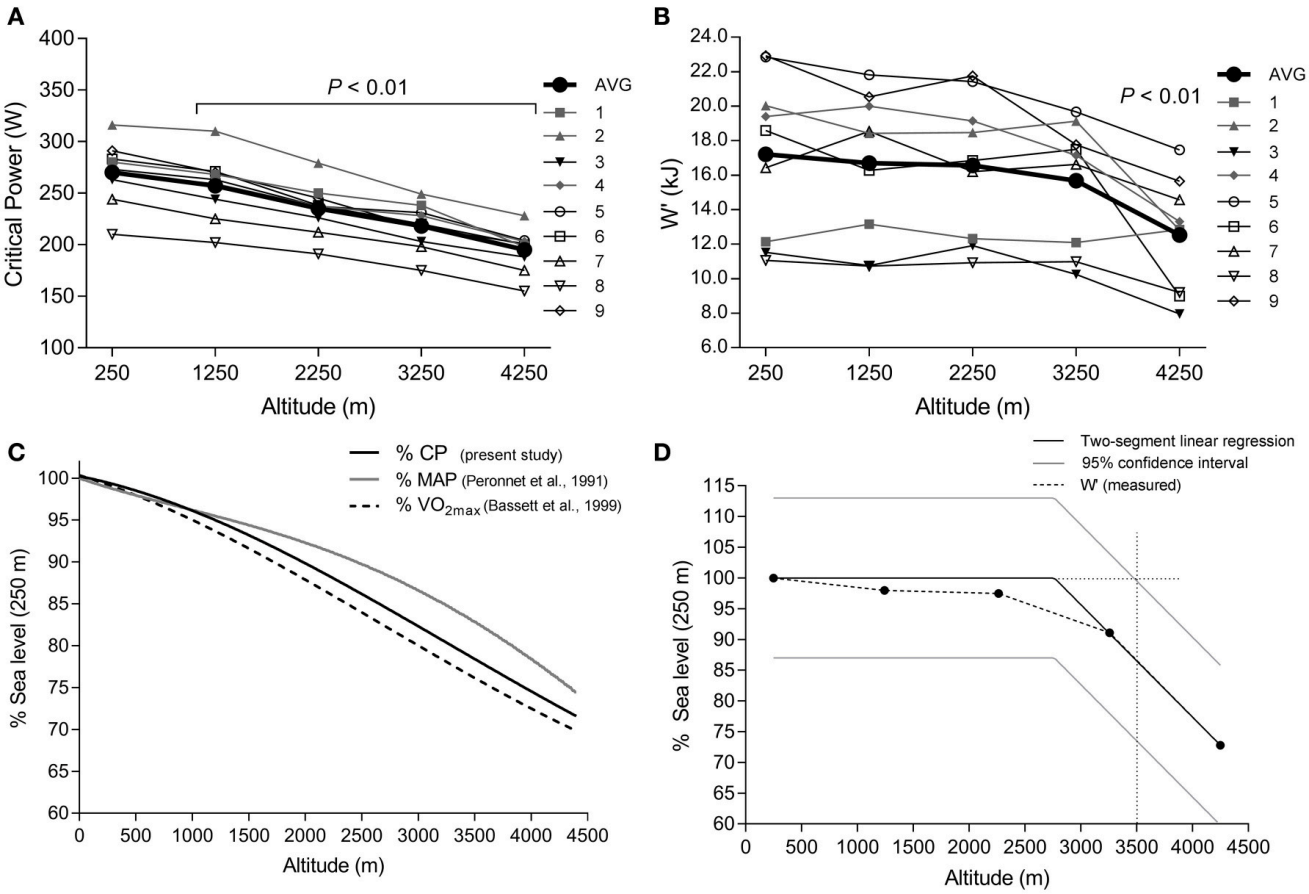
**Effect of increasing altitude on group mean and individual subject critical power (A)** and W′ **(B)**. Model predicted critical power also showing comparison to maximal aerobic power (MAP) and $\dot{\text{V}}$O_2max_ **(C)**, and W′ **(D)**, expressed as percent of sea level measured values. In **(D)** light gray solid lines represent 95% CI. Intersection of the dotted lines indicates predicted altitude where a statistically significant decline in W′ would occur. *P* < 0.05 indicates significant difference compared to 250 m.

Individual and group mean changes at altitude in CP and W′ are presented in Figures 1A,B. At 250 m, mean CP was 269.9 W (95% CI: 250.6, 289.1 W). There was a significant effect of altitude on both CP (*P* < 0.001) and W′ (*P* < 0.001). Compared with 250 m, *post*-*hoc* comparison showed that CP decreased significantly at 1250 m by 13.0 W (95% CI: 5.6, 20.3; *g* = 0.41; *P* < 0.001), at 2,250 m by 34.9 W (95% CI: 24.8, 44.9; *g* = 1.22; *P* < 0.001), at 3,250 m by 52.3 W (95% CI: 39.9, 64.8; *g* = 1.64; *P* < 0.001), and at 4,250 m by 74.0 W (95% CI: 59.7, 90.1; *g* = 2.87; *P* < 0.001). Mean W′ at 250 m was 17.2 kJ (95% CI: 14.3, 20.1 kJ). Compared with 250 m, no significant differences were found at 1,250 m (−0.5 kJ; 95% CI: −1.6, 2.7; *g* = 0.11; *P* = 0.99), 2,250 m (−0.5 kJ; 95% CI: −2.8, 1.7; *g* = 0.12; *P* = 0.99), or 3,250 m (−1.7 kJ; 95% CI: −4.0, 0.7; *g* = 0.39; *P* = 0.3). At 4,250 m W′ was significantly lower than 250 m (−4.7 kJ; 95% CI: −7.1, −2.3; *g* = 1.18; *P* < 0.001).

### Modelling CP and W′ at altitude

Baseline CP at 250 m was correlated with the magnitude of decline in CP at altitude (expressed as ΔW/km altitude. *r* = 0.89; *P* = 0.001). However, when the decline in CP at altitude was expressed as percent changes, this relationship was not significant (*r* = 0.47; *P* = 0.21). Therefore, to simplify the CP prediction equation, we chose to fit the data as percent changes. Using least squares regression, the decrease in CP with increasing altitude (Figure 1C) was best fit to a 3rd order polynomial function (*r*^2^ = 0.99) as follows:

(7) $$\begin{array}{r} y=0.0016x^{3}-0.0157x^{2}-0.027x+1.0025 \end{array}$$

Where *y* = the percent decline in CP from sea level values, and *x* = altitude in km.

The effect of altitude on W′ was described using a two segment linear model, whereby the intercept and gradient of line one were constrained to 100 and 0%, respectively. The gradient for line 2 was −18.3% (per km) and the breakpoint was 2.76 km. Assuming a mean 95% confidence interval range for W′ measures at all altitudes, this two-segment model predicts W′ measured at sea level, to decline significantly beyond ≈3,500 m (Figure 1D).

### ${\text{W}}_{\text{BAL}}^{\prime }$ modelling during intermittent task

**Figure 2:**
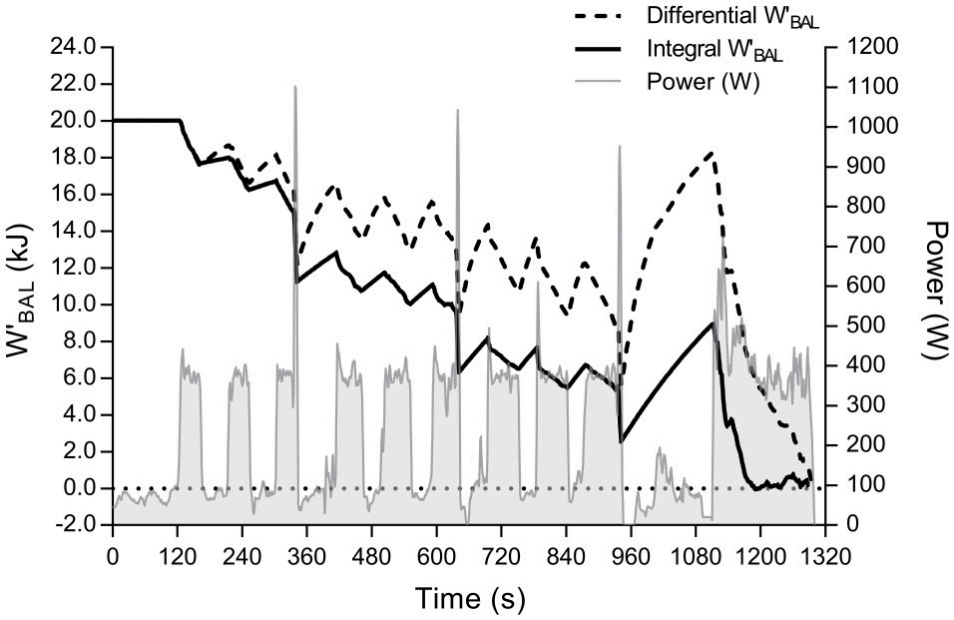
**${\text{W}}_{\text{BAL}}^{\prime }$ responses, showing both integral (solid black line) and differential (dotted line) model computations, during the high intensity intermittent test**. Light gray shaded area shows actual power output.

Figure 2 shows modelled ${\text{W}}_{\text{BAL}}^{\prime }$ during the HIIT for an individual subject. Table 1 presents group mean data for all ${\text{W}}_{\text{BAL}}^{\prime }$ computations. There was no significant effect of parameter input (actual vs. corrected CP and W′) on modelled ${\text{W}}_{\text{BAL}}^{\prime }$ at 2,250 m (*P* = 0.24). A significant main effect of model (${\text{W}}_{\text{BALint}}^{\prime }$ vs. ${\text{W}}_{\text{BALdiff}}^{\prime }$) was observed (*P* < 0.001), and also altitude (250 vs. 2,250 m. *P* < 0.01). The altitude by model interaction was significant (*P* = 0.02), where *post-hoc* comparison revealed a significant effect of altitude for the ${\text{W}}_{\text{BALdiff}}^{\prime }$ model only (−0.6 kJ; 95% CI: −0.8, −0.4; *g* = 0.86; *P* < 0.001).

**Table 1:** **Mean ± SD modelled ${\text{W}}_{\text{BAL}}^{\prime }$ responses at the completion of each interval during the HIIT**.

		**Intermittent task interval number**										
		**Initial W′**	**1**	**2**	**3**	**4**	**5**	**6**	**7**	**8**	**9**	**3TT**
**250 M**												
${\text{W}}_{\text{BALdiff}}^{\prime }$	Actual	17.2 ± 4.7	15.0 ± 4.1	13.7 ± 3.5	13.4 ± 3.3	11.1 ± 2.7	10.6 ± 2.4	10.8 ± 2.3	8.7 ± 2.1	8.0 ± 1.5	7.4 ± 1.3	0.7 ± 2.0
${\text{W}}_{\text{BALint}}^{\prime }$	Actual	17.2 ± 4.7	15.1 ± 4.2	13.5 ± 3.6	12.5 ± 3.3	8.6 ± 2.9^**^	7.8 ± 2.7^**^	7.6 ± 2.6^**^	4.7 ± 2.5^**^	4.4 ± 2.2^**^	4.1 ± 2.0^**^	−0.9 ± 1.3^*^
**2,250 M**												
${\text{W}}_{\text{BALdiff}}^{\prime }$	Actual	16.9 ± 4.0	14.8 ± 3.4	13.6 ± 3.1	13.2 ± 2.9	10.7 ± 2.4	10.2 ± 2.3	10.3 ± 2.2	7.7 ± 2.0^†^	7.0 ± 1.9^†^	6.4 ± 1.8^†^	−1.3 ± 3.5^††^
	Corrected	16.9 ± 4.0	14.8 ± 3.7	13.6 ± 3.4	13.3 ± 3.2	10.7 ± 2.6	10.3 ± 2.6	10.4 ± 2.6	7.9 ± 2.3	7.2 ± 2.1	6.6 ± 2.0	−1.1 ± 3.3
${\text{W}}_{\text{BALint}}^{\prime }$	Actual	16.9 ± 4.0	14.9 ± 3.5	13.4 ± 3.1	12.5 ± 3.0	8.6 ± 2.6^**^	8.0 ± 2.6^**^	7.7 ± 2.6^**^	4.5 ± 2.4^**^	4.1 ± 2.4^**^	3.9 ± 2.3^**^	−2.8 ± 2.8^*^^††^
	Corrected	16.9 ± 4.0	14.9 ± 3.5	13.4 ± 3.2	12.6 ± 3.1	8.7 ± 2.8^**^	8.1 ± 2.9^**^	7.8 ± 3.0^**^	4.6 ± 2.8^**^	4.3 ± 2.7^**^	4.1 ± 2.7^**^	−2.6 ± 2.6^*^

* *P < 0.05 Integral vs. differential*.

** *P < 0.01 Integral vs. differential*.

† *P < 0.05 2,250 m vs. 250 m*.

†† *P < 0.01 2,250 m vs. 250 m*.

**Figure 3:**
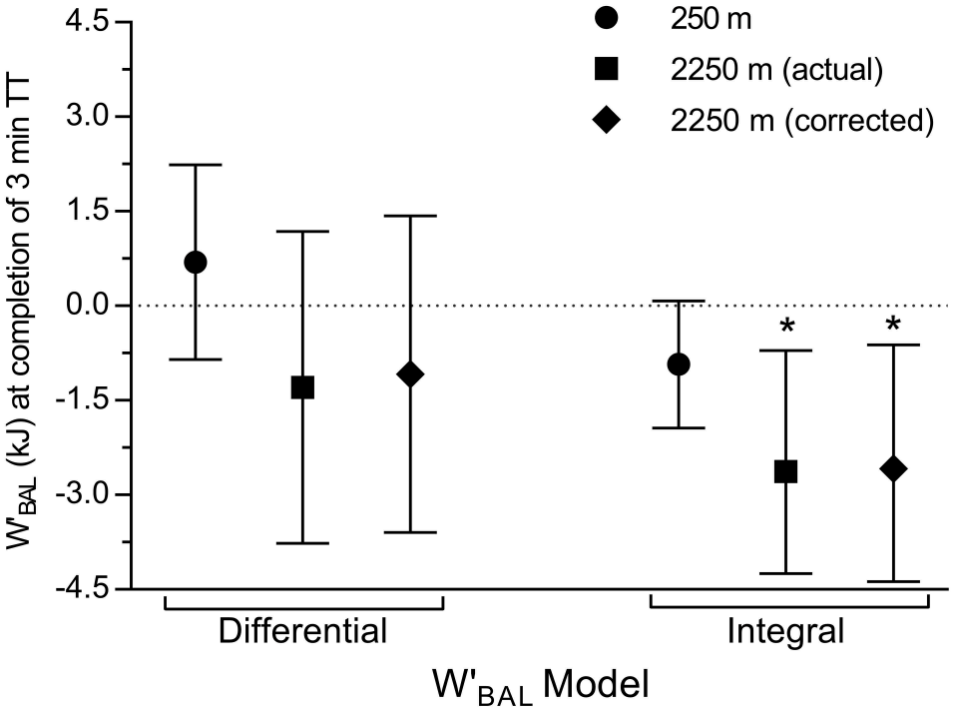
**Modelled ${\text{W}}_{\text{BAL}}^{\prime }$ at completion of the 3TT which concludes the HIIT expressed as mean ± 95% CI**. Dotted line at ${\text{W}}_{\text{BAL}}^{\prime }$ = 0 kJ indicates the theoretical point of exhaustion. ^*^P < 0.05 difference compared with 0 kJ.

**Figure 4:**
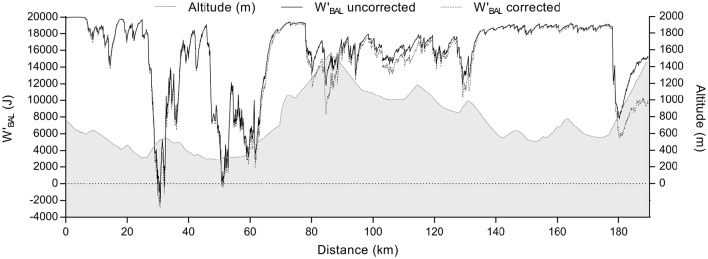
**Field data collected during the 2015 Giro d'Italia showing the effect of altitude correction of CP and W′ on modelled ${\text{W}}_{\text{BALdiff}}^{\prime }$ response**.

Figure 3 displays computed ${\text{W}}_{\text{BAL}}^{\prime }$ at completion of the 3TT (which concludes the HIIT) for all model variants. ${\text{W}}_{\text{BALdiff}}^{\prime }$ was not different to a value of 0 kJ, which theoretically represents the limit of tolerance during high intensity exercise, at either 250 m (0.7 kJ; 95% CI: −0.9, 2.2 kJ; *g* = 0.34; *P* = 0.33), or 2,250 m for actual model inputs (−1.3 kJ; 95% CI: −3.9, 1.4 kJ; *g* = 0.37; *P* = 0.30), and altitude corrected inputs (−1.1 kJ; 95% CI: −3.6, 1.4 kJ; *g* = 0.33; *P* = 0.35). ${\text{W}}_{\text{BALint}}^{\prime }$ was different to 0 kJ at 2,250 m for both actual (−2.8 kJ; 95% CI: −4.9, −0.7 kJ; *g* = 1.03; *P* = 0.02) and altitude corrected inputs (−2.6 kJ; 95% CI: −4.5, −0.6 kJ; *g* = 1.02; *P* = 0.02), whilst the difference approached significance at 250 m (−0.9 kJ; 95% CI: −1.9, 0.04 kJ; *g* = 0.74; *P* = 0.058). An example of a field based practical application of the altitude correction to ${\text{W}}_{\text{BALdiff}}^{\prime }$ is shown in Figure 4, which was computed from field data during the 2015 Giro d'Italia.

## Discussion

Previous studies have quantified the decrease in $\dot{\text{V}}$O_2max_ that occurs at altitude (Bassett et al., 1999; Wehrlin and Hallén, 2006; Clark et al., 2007; MacInnis et al., 2015), however this is the first to examine the effect of altitude on CP and W′. Consistent with data on the dose-response effect of hypoxia on $\dot{\text{V}}$O_2max_ (Bassett et al., 1999; MacInnis et al., 2015), we observed a curvilinear decrease in CP. Secondly, the effect of hypoxia on W′ appears to display threshold type characteristics since there were no significant changes at lower altitudes, whereas a decrease occurred at the highest altitude only (4,250 m). Lastly, we have demonstrated for the first time that a prediction equation can be used in place of actual measurements of CP + W′ at simulated altitude of 2,250 m, to characterize intermittent high-intensity exercise using the ${\text{W}}_{\text{BAL}}^{\prime }$ model.

With increasing altitude, $\dot{\text{V}}$O_2max_ has been shown to decrease linearly (Wehrlin and Hallén, 2006; Clark et al., 2007), or curvilinearly (Bassett et al., 1999; MacInnis et al., 2015). The curvilinear decrease in CP observed in this study (see Figures 1A,C) was similar to that for $\dot{\text{V}}$O_2max_ reported by Bassett et al. (1999). Whilst the effect of hypoxia on the factors that determine $\dot{\text{V}}$O_2max_ are well understood (Wagner, 1996), less is known about the determinants of CP in hypoxia. Traditionally, CP has been considered to reflect a rate limited aerobic energetic supply (Jones et al., 2010). However, it is important to note that $\dot{\text{V}}$O_2_ at CP is below $\dot{\text{V}}$O_2max_ (Poole et al., 1988; Vanhatalo et al., 2016), and therefore oxidative metabolism at CP cannot be “rate limited.” Rather, CP is associated with the highest exercise intensity where a $\dot{\text{V}}$O_2_ steady state, and muscle “metabolic stability” can be achieved (Poole et al., 1988; Jones et al., 2008; Vanhatalo et al., 2016). Metabolic stability is characterized by minimal disturbance to intramuscular [PCr], [P_i_], [H^+^], [ADP_free_], [AMP_free_] and Gibbs free energy of ATP hydrolysis (Grassi et al., 2011). In hypoxia, a decrease in convective O_2_ transport to working muscle occurs (Amann and Calbet, 2008), and the $\dot{\text{V}}$O_2_ primary component decelerates (Hughson and Kowalchuk, 1995). Since the $\dot{\text{V}}$O_2_ primary component is considered an “epiphenomenon” of metabolic stability (Grassi et al., 2011), and has been shown to correlate with CP (Murgatroyd et al., 2011), then an O_2_ supply limitation on $\dot{\text{V}}$O_2_ kinetics may impair metabolic stability, and thus explain why CP is reduced in hypoxia (Dekerle et al., 2012; Simpson et al., 2015; Shearman et al., 2016).

In the present study we found no significant differences in W′ at moderate altitudes up to 3,250 m, however a marked reduction (≈27%) occurred at 4,250 m (see Figure 1B). These results broadly align with several other investigations examining the effect of differing magnitudes of hypoxia on W′ (Valli et al., 2011; Dekerle et al., 2012; Simpson et al., 2015; Shearman et al., 2016). In recent years improved understanding of the mechanistic basis of W′ has developed. When the exercise intensity increases beyond CP, there is progressive recruitment of type IIx muscle fibers (Copp et al., 2010) and a slowing of $\dot{\text{V}}$O_2_ uptake kinetics (Brittain et al., 2001). Slower $\dot{\text{V}}$O_2_ uptake kinetics allows progressive deterioration of muscle metabolic stability to occur which has been demonstrated for both constant load (Jones et al., 2008), and intermittent exercise (Chidnok et al., 2013). The cellular changes associated with failure of metabolic stability are believed to be linked to the emergence of the $\dot{\text{V}}$O_2_ slow component, and to underlie mechanisms of peripheral muscle fatigue (Grassi et al., 2015). Murgatroyd et al. (Murgatroyd et al., 2011) reported a significant correlation between the $\dot{\text{V}}$O_2_ slow component magnitude and W′, which suggests the capacity to complete work above CP until the point of exhaustion is ultimately determined by the mechanisms of fatigue contributing to task failure. In moderate hypoxia (FiO_2_: ≈0.15), the absolute magnitude of peripheral muscle fatigue, assessed via twitch interpolation, remains similar to normoxia following either constant load work to task failure (Amann et al., 2007), or self-paced TT exercise (Amann et al., 2006a). Additionally, Romer et al. (Romer et al., 2007) found the rate of peripheral fatigue development to increase in hypoxia (FiO_2_: ≈0.13) compared with normoxia, but the absolute magnitude remained similar. These findings support the notion that the apparent fixed nature of W′ may be linked to the existence of a peripheral muscle fatigue limit which cannot be surpassed despite varying experimental conditions, including hypoxia (Amann et al., 2006b; Romer et al., 2007; Poole et al., 2016). However, since hypoxia reduces CP, a given absolute workrate in the severe domain corresponds to a higher intensity *relative* to CP, compared with normoxic conditions. Therefore, according to the CP model, W′ should deplete faster and time to exhaustion would decline. This would be associated with an exacerbated rate of fatigue development in hypoxia, as shown by Romer et al. (Romer et al., 2007). Hence, rather than conceptualizing hypoxia *per se* as the mechanism which exacerbates fatigue, it is the effect of hypoxia on decreasing CP that indirectly leads to a more rapid onset of fatigue, coincident with depletion of W′, at a given absolute workrate.

This study is the first to examine the effect of increasing altitude on W′. Our finding that W′ was markedly reduced only in severe hypoxia (≈27% at 4,250 m) led to the construction of a two-segment linear model (see Figure 1D). Using an average confidence interval across all trials, we estimated that a significant decrease in W′ would occur at altitudes beyond ≈3,500 m. Simpson et al. (2015) reported a small decrease in W′ at 3,800 m, but this did not reach statistical significance, whilst Valli et al. (2011) found a large decrease (≈55%) in W′ at 5,050 m. Thus, it appears likely that severe hypoxia reduces W′, yet some uncertainty remains regarding the lowest altitude at which this occurs. Measurement of W′ shows high within-subject variability (Karsten et al., 2014) though, which may confound attempts to accurately determine such a threshold altitude. Valli et al. (2011) suggested the decrease in W′ was consistent with reduced muscle-venous O_2_ storage. More recent evidence reveals a decrease in central motor drive in severe hypoxia (≈5,250 m), but no change in moderate hypoxia (≈2,500 m), compared with sea level (Amann et al., 2007). Group III/IV afferent feedback from the locomotor muscles has been suggested to regulate central motor drive (Amann et al., 2006a, 2007), although evidence also suggests that a direct effect of cerebral hypoxia, independent of afferent feedback, may contribute to reduced performance and altered central motor drive in severe hypoxia (Millet et al., 2012). A direct inhibitory effect of cerebral hypoxia on central motor drive might explain the reduction in W′ found in this study and that of Valli et al. (2011), and also the finding that peripheral fatigue is significantly reduced at task failure only in severe hypoxia, but not moderate hypoxia (Amann et al., 2007).

In order to extend the applicability of the constant load CP model to intermittent high-intensity exercise, Skiba et al. (2012) introduced the ${\text{W}}_{\text{BALint}}^{\prime }$ model. This model includes the following assumptions, (1) expenditure of W′ occurs when the power output exceeds CP, (2) reconstitution of the W′ occurs when the power output falls below CP, and (3) the reconstitution of W′ follows a predictable monoexponential time course. The ${\text{W}}_{\text{BALint}}^{\prime }$ model has been validated empirically in normoxia (Skiba et al., 2012, 2014b), whilst a receiver-operator characteristic analysis found subjective rating of exhaustion to occur when the modelled ${\text{W}}_{\text{BAL}}^{\prime }$ fell below 1.5 kJ (Skiba et al., 2014a). Previously, we demonstrated the ${\text{W}}_{\text{BALint}}^{\prime }$ model to be applicable during intermittent high intensity exercise at ≈2,450 m (Shearman et al., 2016). However, this model was only valid when CP and W′ were also measured at the same FiO_2_ (Shearman et al., 2016). In the present study, we have shown that a predictable decline in CP occurs with increasing altitude up to 4,250 m (Figures 1A,C), and therefore, ${\text{W}}_{\text{BAL}}^{\prime }$ can be calculated in hypoxia using measurements of CP and W′ in normoxia. We found no difference in computed ${\text{W}}_{\text{BALint}}^{\prime }$ or ${\text{W}}_{\text{BALdiff}}^{\prime }$ during intermittent high-intensity cycling at 2,250 m, when either actual measurements of CP and W′ at 2,250 m were used, or predicted values based on measures at 250 m (see Table 1).

A modified version of the ${\text{W}}_{\text{BALint}}^{\prime }$ model (Skiba et al., 2012) was recently published (Skiba et al., 2015). This newer model adopted principles of chemical reaction kinetics and takes the form of a differential equation, hence it was referred to as the ${\text{W}}_{\text{BAL}}^{\prime }$ “differential” model. The advantage of a differential equation is that the time constant of W′ recovery does not require prior fitting to empirical data (Skiba et al., 2015), or estimation from Equation 4. The present study though, is the first to directly compare the ${\text{W}}_{\text{BALint}}^{\prime }$ model (Skiba et al., 2012) vs. the ${\text{W}}_{\text{BALdiff}}^{\prime }$ model (Skiba et al., 2015), within a single subject sample (see Table 1). We found significantly higher values for computed ${\text{W}}_{\text{BALdiff}}^{\prime }$ from the fourth interval onwards during the HIIT, at which point ${\text{W}}_{\text{BALint}}^{\prime }$ had declined by ≈50% from initial W′. This difference can largely be explained by the smaller time constant (hence faster recovery kinetics) observed for ${\text{W}}_{\text{BALdiff}}^{\prime }$ vs. the ${\text{W}}_{\text{BALint}}^{\prime }$ model (Skiba et al., 2015). Upon completion of the 3TT at both 250 or 2250 m, ${\text{W}}_{\text{BALdiff}}^{\prime }$ values displayed only small effect sizes and non-significant differences compared with a theoretical criterion of 0 kJ. However, the difference in ${\text{W}}_{\text{BALint}}^{\prime }$ values was moderate at 250 m and large at 2,250 m. This finding contrasts our previous work (Shearman et al., 2016), in which the ${\text{W}}_{\text{BALint}}^{\prime }$ model showed good agreement with a criterion range of ${\text{W}}_{\text{BAL}}^{\prime }$ = 0 ± 1.5 kJ at task failure. In the present study however, we included all-out sprint efforts in addition to self-paced TT exercise during the HIIT. Whilst the ${\text{W}}_{\text{BALdiff}}^{\prime }$ model appeared better suited to the HIIT in this study, the short recovery time constant led to faster W′ reconstitution than reported by Ferguson et al. (2010) for longer recovery durations. Further research is required to understand the limitations of the current mathematical approaches and to develop a more robust model of intermittent exercise.

Since interval training and road cycling competition is highly stochastic in nature, there are limitless permutations of intensity and work to rest ratios. The application of ${\text{W}}_{\text{BAL}}^{\prime }$ approach though, enables analysis of all such permutations within a single unifying mathematical framework. A key justification for the present study was to extend the practical application of ${\text{W}}_{\text{BAL}}^{\prime }$ during dynamic environmental conditions such as a mountain climb in cycling. Figure 4 presents competition field data from the 2015 Giro d'Italia, during a stage which ascends beyond 1,400 m. The effect of increasing altitude can be seen by comparing the uncorrected CP vs. corrected (for altitude) parameter input into ${\text{W}}_{\text{BAL}}^{\prime }$ model. Interestingly, despite a maximum reported effort on the final hill climb, it appears as though ${\text{W}}_{\text{BAL}}^{\prime }$ is reconstituting since the power is below CP. The failure to deplete ${\text{W}}_{\text{BAL}}^{\prime }$ in this instance likely reflects prolonged accumulation of fatigue mechanisms such as glycogen depletion and/or increasing central fatigue (Thomas et al., 2015), which are not taken into account within the framework of the current models. Glycogen depletion has been shown to decrease the value of W′ (Miura et al., 2000), and fatigue induced inefficiency might decrease CP (Grassi et al., 2015). Accordingly, further research is warranted to develop a robust ${\text{W}}_{\text{BAL}}^{\prime }$ model for a variety of different task requirements. Prolonged endurance exercise would be one such example.

It is well known that endurance exercise performance is reduced upon ascent to altitude (Amann and Calbet, 2008). Secondly, recent progress has been made in the field of mathematical modelling intermittent high-intensity performance, which has practical applications in training load prescription and monitoring (Skiba et al., 2014a, 2015; Shearman et al., 2016). In the present investigation, we report a curvilinear decrease in CP with increasing altitude as well as a significant reduction in W′ occurring only at 4,250 m. The predictable decline in CP, combined with lack of change in W′ up to 3,250 m, enables modelling of ${\text{W}}_{\text{BAL}}^{\prime }$ in hypoxic environments without the requirement for testing at all altitudes. This enables the prescription of equivalent relative intensity interval training workouts in hypoxic conditions compared with normoxia. Whilst we validated use of the altitude correction factor within the ${\text{W}}_{\text{BAL}}^{\prime }$ at 2,250 m, since it is known that W′ contains relatively high typical error (Karsten et al., 2014, 2016), and there may be changes in W′ at higher altitudes, caution is required when interpreting modelled intermittent performance in severe hypoxia above ≈3,500 m.

## Author contributions

NT, DN, PS, and JP contributed to experimental concept and design. NT, DN, SR, and JP contributed to data collection and analysis. NT, PS, SR, and JP contributed to manuscript preparation. The authors declare that the results of the study are presented clearly, honestly, and without fabrication, falsification, or inappropriate data manipulation.

### Conflict of interest statement

The authors declare that the research was conducted in the absence of any commercial or financial relationships that could be construed as a potential conflict of interest.

## Abbreviations

P: Power
W: Watt
CP: Critical power; asymptote of the power–duration relationship
W′: “W-prime” curvature constant of the power–duration relationship
t_lim_: Time to exhaustion
${\text{W}}_{\text{BAL}}^{\prime }$: Work-balance: Amount or balance of W′ remaining
${\text{W}}_{\text{BALint}}^{\prime }$: Integral equation form of ${\text{W}}_{\text{BAL}}^{\prime }$ model
${\text{W}}_{\text{BALdiff}}^{\prime }$: Differential equation form of ${\text{W}}_{\text{BAL}}^{\prime }$ model
$\tau_{\text{W}}^{\prime }$: Time constant for reconstitution of W′
PCr: Intramuscular phosphocreatine
P_i_: Intramuscular inorganic phosphate
H^+^: Intramuscular hydrogen ion
ADP_free_: Intramuscular adenosine di-phosphate free
AMP_free_: Intramuscular adenosine mono-phosphate free
O_2_: Oxygen
$\dot{\text{V}}$O_2_: Volume of oxygen consumption
HIIT: High-intensity intermittent test
3TT: 3 min time trial
FiO_2_: Fraction of inspired oxygen.
